# Hysterectomy with Fetus In Situ for Uterine Rupture at 21-Week Gestation due to a Morbidly Adherent Placenta

**DOI:** 10.1155/2018/5430591

**Published:** 2018-09-02

**Authors:** Katerina Pizzuto, Cory Ozimok, Radenka Bozanovic, Kathleen Tafler, Sarah Scattolon, Nicholas A. Leyland, Michelle Morais

**Affiliations:** ^1^School of Medicine, McMaster University, 1280 Main St. West, Hamilton, Ontario L8S4K1, Canada; ^2^Department of Obstetrics and Gynecology, McMaster University, 1280 Main St. West, Hamilton, Ontario L8S4L8, Canada; ^3^Department of Radiology, McMaster University, 1200 Main St. West, Hamilton, Ontario L8N3Z5, Canada; ^4^Department of Pathology, McMaster University, 1200 Main St. West, Hamilton, Ontario L8N 3Z5, Canada

## Abstract

**Background:**

Uterine rupture due to a morbidly adherent placenta is a rare obstetrical cause of acute abdominal pain in the pregnant patient. We present a case to add to the small body of published literature describing this diagnosis.

**Case:**

A 32-year-old G5T2P1A1L2 with multiple prior cesarean sections presented at 21^+3^ weeks' gestation with abdominal pain and presyncope. Ultrasound showed a large volume of complex intraabdominal free fluid and a heterogenous placenta with irregular lacunae and increased vascularity extending to the posterior bladder wall. Exploratory laparotomy identified a uterine defect and a hysterectomy was performed due to significant bleeding. Pathology confirmed a diagnosis of placenta percreta.

**Conclusion:**

Early recognition and management of uterine rupture due to a morbidly adherent placenta are essential to prevent catastrophic hemorrhage.

## 1. Introduction

Pregnant patients commonly present with abdominal pain. Diagnosis can be challenging as the differential for both obstetric and nonobstetric causes can be extensive, and the physical examination can be altered when a gravid uterus is present. Two rare obstetric causes of acute abdominal pain include uterine rupture and intra-abdominal hemorrhage due to a morbidly adherent placenta. As the rate of cesarean sections increases, these severe complications may become more frequent and should be included in the differential diagnosis for abdominal pain in pregnancy. This case report aims to contribute to the small body of published literature describing these rare complications of pregnancy.

## 2. Case

A 32-year-old G5T2P1A1L2, at 21 weeks and 3 days of gestation, was brought to Labour and Delivery Triage at a tertiary care centre by ambulance. The patient had noted abdominal pain that she felt may be bowel related. After attempting to have a bowel movement, she experienced a presyncopal episode that prompted her to call for an ambulance.

Upon arrival, the maternal heart rate was 71, respiratory rate was 18, oxygen saturation was 98% on room air, and blood pressure was 80/40 mmHg. The fetal heart rate was auscultated to be normal at 145 beats per minute. The patient arrived with an intravenous line in situ and was receiving a fluid bolus. She appeared to be in pain but was awake and oriented. On history, the patient did not endorse any change to her bowel habits, fever, nausea, or vomiting. She did not have any vaginal bleeding, contraction-like pain, rupture of membranes, or abnormal vaginal discharge. Her past obstetrical history was significant for a therapeutic abortion, a classical cesarean section for a stillborn infant after preterm premature rupture of membranes and cord prolapse, an elective cesarean section at term, and a subsequent elective cesarean section at term with an incidental finding of uterine dehiscence at the time of surgery. She was otherwise healthy.

In her current pregnancy, she had been referred to the Maternal Fetal Medicine service for investigation of a suspected abnormally adherent placenta, possibly placenta increta or placenta percreta. This was identified at the time of her anatomy ultrasound, when a complete anterior placenta previa was noted, along with concerning findings including loss of the placental-myometrial interface, multiple large and irregularly shaped lacunae, significant vascularity within the myometrium abutting the bladder wall, and a marginal placental abruption measuring 37.5 x 57.6 x 9.5mm. The fetus was appropriately grown and anatomic survey was normal.

Repeat vital signs continued to be stable. Blood pressure improved to 92/51 with an ongoing fluid bolus. The abdomen was soft and tender throughout, with most pain in the right lower quadrant; however, the uterus itself was nontender. In addition, there was rebound tenderness and voluntary guarding.

**Figure 1:**
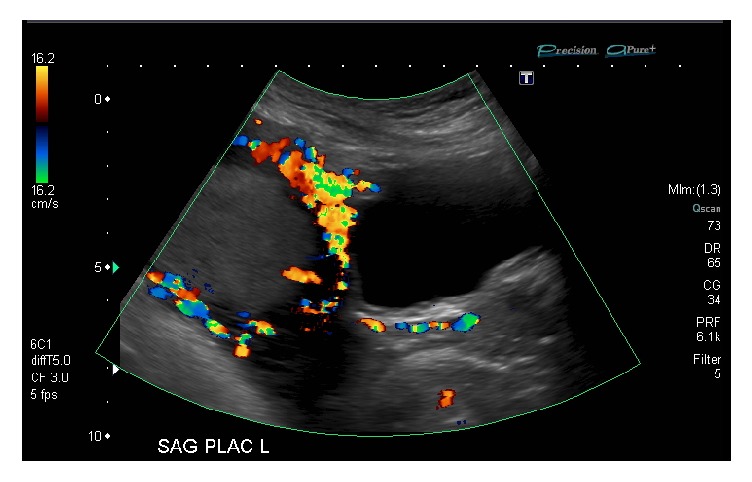
Doppler ultrasound in the sagittal plane at midline in the pelvis demonstrates turbulent peripheral vascularity in the placenta extending across the myometrium to the posterior wall of the bladder. The bladder contour is otherwise smooth; however, the finding remains highly suggestive of placenta percreta. No defect in the uterine wall could be identified, but given the large volume of hemorrhagic ascites, an emergency diagnostic laparoscopy was subsequently performed.

Laboratory investigations initially revealed a hemoglobin of 87 g/L, leukocytes of 12.0 x 10^9^ per liter, and platelets of 154 x 10^∧^9/L. AST was 13 U/L ALT 7 U/L and Kleihauer-Betke was negative. The patient was known to be anemic, with a hemoglobin of 92 g/L approximately one month prior. An abdominal ultrasound done emergently identified a normal liver, biliary tree, common bile duct, spleen, aorta, kidneys, and gallbladder. There was no evidence of hydronephrosis or renal stone. The appendix was not visualized. There was no evidence of ovarian torsion. There was free fluid throughout the abdomen with low-level echoes concerning for blood. In the left flank there was a large heterogeneous mass-like echogenic area without internal vascularity, measuring 14 x 10 cm, concerning an intra-abdominal hematoma. An obstetrical ultrasound identified a single live intrauterine gestation. The placenta was again noted to be heterogeneous with multiple irregularly shaped lacunae, and the border between the myometrium and placenta was difficult to identify along the anterior wall, as shown in Figure 1. There was increased vascularity at the placental base and along the myometrial-bladder interface. Attempts were made to assess the integrity of the uterine wall, but visualization was limited due to the fluid in the extrauterine spaces of the pelvis. There was no clear area of uterine dehiscence identified.

Blood work was repeated several hours after the initial measurements, once the suspicion of intra-abdominal hemorrhage was reported on ultrasound. Repeat hemoglobin was stable at 87 g/L, leukocytes rose to 15.6 g/L and platelets were relatively stable at 144 x 10^∧^9/L. Coagulation studies showed a normal INR and PTT of 0.9 and 30 s, respectively, but fibrinogen was relatively low for the pregnancy state at 2.7 g/L. There was no further deterioration of clotting factors throughout the intraoperative course. The patient's vital signs remained stable, with continued slightly low blood pressure. Despite the stable hemoglobin, given the ultrasound findings suggestive of intra-abdominal hemorrhage and hematoma, along with the clinical finding of an acute surgical abdomen, the patient was taken to the operating room for an exploratory laparotomy via Pfannenstiel incision. The main concern was for bleeding due to either uterine rupture and/or bleeding from placenta percreta. The patient was counseled extensively regarding this possibility and the current previable gestation. Consent was obtained to proceed with hysterectomy if these concerns were confirmed intraoperatively.

Upon entry into the peritoneal cavity there was a significant amount of old and new blood, which was immediately evacuated. A small defect on the anterior surface of the uteruswas actively bleeding; it was felt to be the source of the hemoperitoneum. This focal area of the uterine wall was very thin, revealing placenta extending through the level of the serosa. Internal iliac ligation and hysterectomy were performed with the fetus in situ, due to the active bleeding. The estimated blood loss was 2.5 liters, most of which was noted in the abdomen at the beginning of the surgical procedure from prior blood loss. The patient received 3 units of packed red blood cells, 2 units of fresh frozen plasma, and 10 units of cryoprecipitate and 1L of Ringer's Lactate intraoperatively.

**Figure 2:**
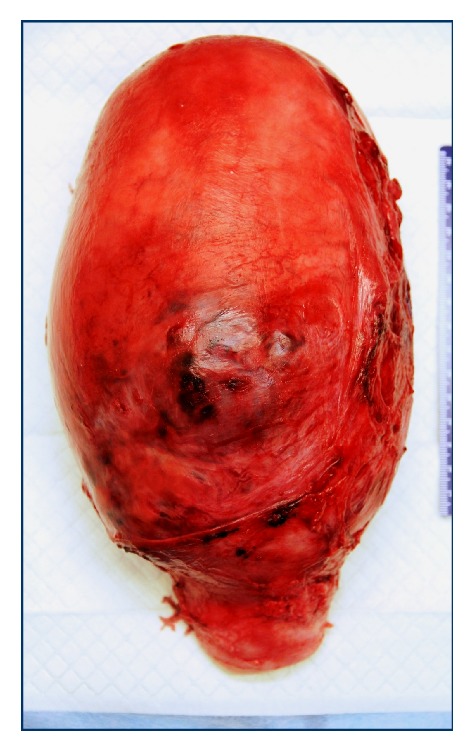
Intact hysterectomy specimen. Anterior wall demonstrates softened tumescence, with patchy hemorrhagic and congested appearance.

Fresh surgical specimens were forwarded to pathology, including bilateral fallopian tubes, the cervix, and the uterus containing the fetus and placenta. The uterine cornua and fallopian tubes, as well as peritoneal reflections, were anatomically normally in position. The serosal surface was intact, except for a 1.0 x 0.6 cm variegated, slightly ragged area, at the midline of the anterior wall, approximately equidistant from the fundus and cervix (Figure 2).

A subchorionic blood clot, continuous with a retroplacental hemorrhage, was noted. A brick-like color indicated that bleeding was remote. Cross-sections of the uterus demonstrated placental infiltration directly under the serosa, with a variegated appearance of the clotted blood admixed with infarcted tissue. Representative sections from the deepest point of placental invasion demonstrated retroplacental hematoma directly abutting serosal uterine surface with adjacent nonviable villi. Overall gross and microscopic findings confirmed the clinical diagnosis of placenta percreta with retroplacental hematoma.

## 3. Discussion

Placenta accreta is defined as an abnormally adherent placenta that invades and is inseparable, from the uterine wall. The term placenta increta is used when the chorionic villi invade only the myometrium, whereas placenta percreta describes invasion through the myometrium and serosa and occasionally into adjacent organs [1]. The incidence of placenta accreta has been steadily increasing over time and is approximately 1 in 533 pregnancies [2]. Several risk factors have been identified for the development of placenta accreta, including previous cesarean delivery, advanced maternal age, high gravidity, multiparity, previous uterine curettage, and placenta previa [3]. This patient had several of the above-listed risk factors. Placenta accreta is most commonly associated with hemorrhage in the third stage of labour, and there is a high incidence of significant postpartum hemorrhage necessitating hysterectomy [3, 4].

Accurate prenatal diagnosis of placental accreta is vital in order to facilitate appropriate antenatal management, delivery planning, and appropriate patient counseling. Ultrasound is the standard modality for assessing the placenta, but MRI has also proven useful [5]. The normal placenta in the second trimester is homogeneous in echotexture and is separated from the more hypoechoic myometrium by a thin subplacental clear space [6]. Doppler imaging demonstrates regular continuous retroplacental myometrial blood flow. Findings on ultrasonography that can be seen in the spectrum of morbidly adherent placenta include thinning of the myometrium, loss of the subplacental clear space, disruption of the serosa-bladder wall interface, presence of an exophytic mass beyond the uterine serosa, increased retroplacental vascularity, increased vascularity along the bladder wall, and placental lacunae [7]. A heterogenous appearing placenta due to the presence of lacunae, which appear as vascular structures demonstrating turbulent flow on Doppler, has the highest sensitivity, ranging from 78 to 93% after 15-week gestational age. Specificity of this finding is only 78.6% [6]. MRI can be useful in equivocal cases and in the setting of a posterior placental position [5]. Findings diagnostic of placenta accreta include abnormal uterine bulging, heterogenous signal intensity, and T2 dark intraplacental bands [6]. In cases of percreta, placental tissue may be seen extending across the myometrium into surrounding structures; tenting of the bladder wall is highly suggestive of invasion. It has been shown that, although less sensitive compared to US, MRI offers the advantage of more accurate determination of the depth and topography of placental invasion [8]. Many authors advocate a two-step approach for assessing high risk patients, beginning with a second trimester ultrasound at 18-20 weeks and further evaluation with MRI if there are suspicious or inconclusive findings [6, 7, 9].

Uterine rupture or intra-abdominal hemorrhage prior to delivery is a rare complication of placenta accreta. This is the first Canadian case report describing uterine rupture associated with a morbidly adherent placenta in the second trimester. This is also the first reported case of a hysterectomy being performed with fetus in situ for uterine rupture. Unfortunately, our patient presented at a gestational age remote from viability. Prior to surgery, a long discussion with the patient revealed that she did not wish conservative treatment, but rather she preferred definitive management in the case of intra-abdominal hemorrhage due to either uterine rupture or placenta percreta. Due to active hemorrhage intraoperatively, in order to preserve maternal health and well-being and in accordance with the patient's wishes, we felt it was most prudent not to attempt conservative management to prolong the pregnancy. Conservative measures could be considered with guarded optimism in patients wishing to attempt pregnancy preservation.

There is only one case reported by Aboulafia et al. that describes successful conservative management of a patient with a similar presentation [8]. This patient presented at 23 weeks placental tissue protruding through the serosa; thus, the uterus was oversewn to cover the exposed placental tissue. She was managed as an inpatient with daily nonstress tests and the pregnancy was successfully prolonged until 32 weeks when she underwent an elective cesarean section and curettage to remove the densely adherent placenta and no hysterectomy was required [10]. The two other cases of attempted conservative management were unsuccessful. In the case described by Hibczuk, there was a uterine defect identified with placental tissue visible through the uterine serosa, and this area was oversewn and no hysterectomy was performed [11]. This patient clinically deteriorated and required a subsequent hysterectomy. Similarly, in the case reported by Hornemann, an area of extrauterine placental tissue and bleeding was identified and coagulated [8]. Postoperatively, the patient continued to have ongoing hemorrhage and was taken back for a hysterectomy. Based on this literature, we concluded that conservative measures may be ineffective to control bleeding and would possibly leave the uterus and placenta in a compromised condition to support an ongoing pregnancy. Given our patient's wishes for definitive management and the finding of active bleeding from the placental site, we felt it prudent to proceed with hysterectomy as planned preoperatively.

In terms of definitive management, a hysterectomy is most commonly performed. Given that significant hemorrhage can occur during this procedure, adjunct procedures can be used to minimize blood loss including internal iliac balloon occlusion and internal iliac ligation. Internal iliac artery occlusion with a balloon has been shown to significantly reduce the blood loss and risk of hysterectomy in patients undergoing nonemergency cesarean section for morbidly adherent placenta [12]. In an emergency setting, it may be challenging to coordinate a balloon occlusion preoperatively, and this service may not be available in all centres. A surgical alternative to balloon occlusion is internal iliac ligation. Camuzcuoglu et al. describe several cases where internal iliac ligation is helpful and safe in the surgical management of patients with placenta previa and/or percreta to reduce blood loss and risk of reoperation [13]. Historically, internal iliac ligation used to be reserved for intractable bleeding refractory to other surgical management, but its utility has expanded to be used both prophylactically and therapeutically in a number of clinical scenarios with excessive hemorrhage including the treatment of uterine atony, invasive placentation, and uterine laceration [14]. The rare complications of internal iliac ligation can include injury to the iliac vein or ureter, inadvertent external iliac artery ligation, and the development of vesical, perineal, or gluteal necrosis [15]. We do recognize that there is a failure rate of internal iliac ligation and that it can be unsuccessful at stopping hemorrhage in some cases [16]. In this subset of patients, one may consider internal aortic compression as a means of temporizing blood loss while hysterectomy is performed [17]. In this patient, she did have significant blood loss documented at 2.5L, but much of the blood loss occurred prior to the hysterectomy from the area of rupture and can be attributed to the preexisting intraabdominal hemorrhage. In our experience, during cases where there is a significant amount of blood in the operative field and ongoing active bleeding, an internal iliac ligation performed at the outset of the procedure can decrease active bleeding, clearing the operative field while allowing the surgeon to proceed with the remainder of the procedure more expeditiously.

In summary, there have been case reports describing pregnant patients presenting with acute onset of abdominal pain and a surgical abdomen found to have uterine rupture due to morbidly adherent placenta. This is the first Canadian case report, and the first report to describe a hysterectomy performed with fetus in situ where the diagnosis of hemorrhage due to uterine rupture and morbidly adherent placenta was suspected preoperatively. It is important to consider this rare but morbid and severe diagnosis when seeing an obstetrical patient with acute abdominal pain.
